# UDP-Induced Phagocytosis and ATP-Stimulated Chemotactic Migration Are Impaired in* STIM1*^−*/*−^ Microglia In Vitro and In Vivo

**DOI:** 10.1155/2017/8158514

**Published:** 2017-02-15

**Authors:** Hye Min Lim, Heo Woon, Jung Woo Han, Yoshihiro Baba, Tomohiro Kurosaki, Min Goo Lee, Joo Young Kim

**Affiliations:** ^1^Department of Pharmacology and Brain Korea 21 PLUS Project for Medical Science, Yonsei University College of Medicine, Seoul 120-752, Republic of Korea; ^2^Laboratory for Lymphocyte Differentiation, WPI Immunology Frontier Research Center (IFReC), Osaka University, Suita, Osaka 565-0871, Japan; ^3^Laboratory for Lymphocyte Differentiation, RIKEN Research Center for Allergy and Immunology, 1-7-22 Suehiro-cho, Tsurumi-ku, Yokohama, Kanagawa 230-0045, Japan

## Abstract

STIM1 is the only currently known intracellular calcium sensor that functions as the calcium influx regulator controlling immune cell activation. STIM1 function in immune cell calcium signalling has been studied extensively; however, its role in microglia, innate immune cells in brain, has not been fully understood. Here, we report that* STIM1*^−*/*−^ murine microglia lost store-operated calcium influx and displayed aberrant immunological functions. Microglial functions regulated by chronic and global [Ca^2+^]_i_ changes were reduced significantly, including cytokine releases and opsonin-dependent phagocytosis. More dramatically, cellular functions governed by Ca^2+^ regulation in local microdomains at the cell periphery, such as UDP-induced phagocytosis and ATP-stimulated chemotactic migration, were severely reduced in* STIM1*^−*/*−^ microglia. Interestingly, UDP-induced Orai1 mobilization to the peripheral region was greatly attenuated in* STIM1*^−*/*−^ microglia. Their chemotactic migration defect was reproduced in vivo in embryonic brain; the aggregated number of* STIM1*^−*/*−^ microglia in LPS- (lipopolysaccharide-) injected lesions was much smaller than that in wild-type microglia. Furthermore, the neuron phagoptosis activities of activated microglia were significantly diminished in the* STIM1*^−*/*−^ microglia. These in vitro and in vivo results suggest that STIM1-mediated store-operated calcium entry is important for the regulation of global [Ca^2+^]_i_ changes which differentiates into active immune state of microglia, but it is more crucial for the regulation of local [Ca^2+^] microdomains which mediates the acute motility of murine microglia.

## 1. Introduction

Microglia are resident macrophages in the brain that orchestrate inflammatory responses. Microglia detect infection sites, foreign material, and pathological alterations in the brain [1, 2]. Pathological stimuli such as lipopolysaccharide (LPS) activate microglia and induce several morphological changes, including phagocytosis to remove cellular debris, and secretion of chemical mediators such as proinflammatory cytokines, which propagate immunological activities [1]. LPS, a representative immune function inducer of microglia, binds to the TLR4 receptor (toll-like receptor 4) in microglia and induces cytokine secretion and phagocytosis, which is dependent on a sustained increase of intracellular Ca^2+^ concentration ([Ca^2+^]_i_) [3–5]. LPS also induces a shift in the microglial gene expression profile, which is mainly mediated by the NFAT (Nuclear factor of activated T-cell) transcription factor [6]. Activated microglia have different gene expression profiles, secrete cytokines such as TNF-alpha and IL-6 and have the enhanced opsonin-dependent phagocytotic activity [6]. Chronic elevation of [Ca^2+^]_i_ boosts phagocytosis and migration via optimizing the related gene expressions, but UDP-mediated phagocytosis and ATP-induced chemotactic migration are primarily regulated by local [Ca^2+^] in focal microdomains at the edge of cells, such as the phagosome or focal adhesion spot [7–9]. Injured neuronal cells leak nucleotides, which activate several P2 receptors in microglia [2, 10, 11]. P2X_4_ and P2X_7_, the most widely expressed P2X subunits on microglia, are activated by ATP and form a homomeric Ca^2+^-permeable channel [12, 13]. Microglia also express P2Y receptors; particularly P2Y_6_ and P2Y_12_ are activated by UDP, then change [Ca^2+^]_i_ via G_q_-coupled signalling pathways, and cause Ca^2+^ influx from the plasma membrane [8, 10].

It is well known that Ca^2+^ influx through store-operated calcium entry (SOCE) mediates sustained calcium increases during activation of immune functions in microglia [5, 14]. Recently, STIM1 (stromal interaction molecule 1) and STIM2 (stromal interaction molecule 2) have been identified as the only protein family that functions simultaneously as an intracellular calcium storage sensor and as a regulator of calcium influx from the extracellular environment. STIM1 is a type 1, single-spanning membrane protein with an EF hand Ca^2+^-binding motif, which functions as the sensor of ER (Endoplasmic reticulum)-luminal Ca^2+^ [15, 16]. Depletion of ER-luminal Ca^2+^ stores induces a conformational change in STIM1; then, the C-terminal transmembrane domain binds and activates the plasma membrane store-operated channel [SOC; Orai; or transient receptor potential channel (TRPC)], which induce Ca^2+^ influx [17, 18]. However, Ca^2+^ influx through SOCE in microdomains was not studied in microglia.

In the present study, we investigate the function of STIM1-mediated SOCE in pathogen- or purinergic-mediated microglial immune function using* STIM1*^−*/*−^ mice, which have complete depletion of STIM1. Experiments using primary cultured microglia indicated that innate immune functions governed by chronic and global [Ca^2+^]_i_ changes, such as cytokine release and opsonin-dependent phagocytosis, were reduced significantly in* STIM1*^−*/*−^ microglia. Above all, functions regulated by the local [Ca^2+^] microdomains at the edge or periphery of cells, such as UDP-induced phagocytosis and ATP-stimulated chemotactic migration were essentially abolished in* STIM1*^−*/*−^ microglia. The migration impairments of* STIM1*^−*/*−^ microglia were also observed in the embryonic brain in vivo. The number of aggregated* STIM1*^−*/*−^ microglia into the LPS-injected lesion for 24 hours was significantly lower than the number of aggregated WT microglia into the LPS-injected lesion. Furthermore, the neuron phagocytotic activity of microglia had significantly diminished in* STIM1*^−*/*−^ microglia. These in vitro and in vivo results suggest that STIM1 is crucial for the regulation of local [Ca^2+^] microdomains in peripheral region of microglia which mediates the process of acute motility of murine microglia.

## 2. Materials and Methods

### 2.1. Mice

*STIM1* ^*+/*−^ heteromice (C57BL/6 background) were generated as previously described [19, 20]. The mice were bred and maintained according to the Yonsei Medical Center animal research requirements, and all procedures were approved by the Committee on Animal Research at Yonsei Medical Center (protocol number 2011-0112). Since* STIM1*^−*/*−^ mice die within 1 day after birth due to defect in the circulatory system, 17 day embryos taken off from pregnant* STIM1*^*+/*−^ females mated with* STIM1*^*+/*−^ male mice were used to purify each WT and* STIM1*^−*/*−^ microglia. For the microglial migration assays in vivo, the brains of the same aged embryos were used. Genomic DNA isolated from the embryo tail was used for genotyping. Genomic DNA for genotyping was extracted using prepGEM Tissue (PTI0200; ZyGEM, Hamilton, New Zealand).

### 2.2. Isolation and Culture of Primary Mouse Microglial Cells, Astrocytes, and Neurons

Primary cultures of mouse microglia were prepared from the whole brain of an embryonic day 17 (E17) mouse. Each embryo tail was used for genotype analyses to identify WT and* STIM1*^−*/*−^ microglia. Primary mixed cells that had been chemically and mechanically dissociated of the whole brain were seeded in DMEM/F12 (1 : 1) culture medium. Cells were cultured at 37°C in an incubator (Thermo Fisher Scientific, Marietta, OH, USA) humidified with 5% CO_2_. Cell confluency was achieved after 3~4 weeks. After 4 weeks, the primary mixed cells of WT and* STIM1*^−*/*−^ were used a mild trypsinization and shaking method [19]. The upper cell layer of mixed cells was removed in one piece by treating with trypsin 0.25% (1x) solution (SH30042.01; Hyclone Laboratories, Thermo Scientific, Logan, UT, USA) diluted 1 : 4 in DMEM/F12 (1 : 1) culture medium (Dulbecco's modified Eagle medium, 11320-033; Gibco, Life Technologies™, Carlsbad, CA, USA) containing 10% fetal bovine serum (26140-079, Gibco, Life Technologies, Carlsbad, CA, USA) and 1% penicillin-streptomycin (15140-122; Gibco, Life Technologies, Carlsbad, CA, USA) for 20 min at 37°C. The trypsin diluted 1 : 4 in DMEM/F12 was suctioned and replaced with 12 mL of new culture medium. The remained pure microglial cells were attached to the bottom of the T75 flask and were isolated by shaking the T75 flasks of WT,* STIM1*^−*/*−^ at 120 rpm (SLOS-20; Seoulin Bioscience, Seoul, Korea) at 37°C for 1 h. 12 mL of each medium were collected from each flasks, placed a cell strainer with 40 *μ*m pore size (352340; BD Falcon, San Jose, CA, USA), filtered into a fresh 15-mL conical tube, and centrifuged at 4,000 rpm at room temperature for 5 min. Glial cells were grown at a high density into 75 T flasks to extract pure microglia at 7 to 9 days in vitro. After isolate microglia, the remaining cells were maintained in astrocyte-conditioned medium and remaining microglia were depleted by adding 50 mM L-leucine-methyl ester (LME, Sigma Aldrich, St. Louis, MO, USA) for 4 hours. To culture the cortical neuron, the culture of cortical neuron was prepared from 1 day postnatal pups (C57BL/6 strain) as described previously [1] with some modifications. The cerebella were dissociated in Versene solution (1 : 5000) and plated at 0.5 × 10^6^ cells/cm^2^ in 24-well plates coated with Laminin (10 *μ*g/mL in serum-free DMEM). Cells were incubated in Dulbecco's modified Eagle's medium (DMEM) supplemented with heat-inactivated horse serum (5%), fetal calf serum (5%), 13 mM glucose, 0.5 mM HEPES buffer, 25 mM KCl, and 2 mM L -glutamine. Cells were maintained at in a humidified atmosphere of 37°C, 5% CO^2^/95% air. To inhibit growth of nonneuronal cells, 7.5 *μ*M Ara-C (cytosine-D-arabinoside, Sigma Aldrich, St. Louis, MO, USA) was treated to the medium 48 h after plating. Cortical neuronal cells were used at 9 days after primary culture in experiment to ensure morphological and physiological maturity. Cells purities were checked with immunostaining of anti-GFAP antibody, anti-Iba1 antibody, and MAP antibody for glial cells, microglia, and neuron, respectively. The numbers of cells were counted with haemocytometer (Marienfeld, Lauda-Königshofen, Germany).

### 2.3. Ca^2+^ Influx Measurements in* STIM1*^*−/−*^ Mice

Ca^2+^ influx was measured by recording [Ca^2+^]_i_ with Fura-2-AM labelling (F1201; Invitrogen, Molecular Probes, Eugene, Oregon, USA) as described previously [17]. Briefly, WT and* STIM1*^−*/*−^ microglia grown on glass cover slips were loaded with cell-permeable Fura-2-AM, and trapped Fura-2 fluorescence was measured with a spectrofluorometer (Photon Technology International, Birmingham, NJ, USA). Cells were perfused with a solution containing 150 mM NaCl, 5 mM KCl, 1 mM MgCl_2_, 10 mM glucose, 10 mM HEPES (pH 7.4 adjusted with NaOH) containing either 1-2 mM CaCl_2_ or 0.5 mM EGTA (to chelate any Ca^2+^ in the solution). The osmolality of all solutions was adjusted to 310 Osm with the major salt. The Fura-2 ratio was recorded using dual-excitation wavelengths at 340 and 380 nm, and emission wavelengths above 510 nm were monitored. Cells were treated with 25 *μ*M cyclopiazonic acid (CPA; C1530, Sigma Aldrich, St. Louis, MO, USA), which inhibits the endoplasmic reticulum (ER) Ca^2+^-ATPase. Cells were treated with 100 *μ*M uridine 5′-(trihydrogen diphosphate) sodium salt (UDP; U4125, Sigma Aldrich, St. Louis, MO, USA) or 50 *μ*M adenosine 5′-triphosphate di(tris) salt hydrate (ATP; A9062, Sigma Aldrich, St. Louis, MO, USA).

### 2.4. Immunocytochemistry

Primary cultured WT and* STIM1*^−*/*−^ microglia were seeded on glass cover slips into 24-well plates at 5 × 10^4^ cells/well and incubated at 37°C in an incubator enriched with 5% CO_2_ atmosphere overnight. Cells were treated with or without 1 *μ*M thapsigargin (TG; T9033, Sigma Aldrich, St. Louis, MO, USA) for 5 min, fixed with 4% paraformaldehyde in phosphate-buffered saline (PBS) (19943 1 LT; Affymetrix, Cleveland, Ohio, USA) at room temperature for 10 min, and then rinsed three times with PBS. Cells were permeabilized by incubating in permeabilization solution [0.1% Triton X-100, 1% horse serum, and 1% bovine serum albumin (BSA) in PBS, pH 7.4] at room temperature for 10 min, and then rinsed three times with PBS. To avoid nonspecific antibody binding, cells were treated with blocking solution (5% horse serum, 1% BSA, 0.1% gelatin, and 0.001% sodium azide in PBS, pH 7.4) at room temperature for 30 min. Immunostaining was performed in blocking solution using 1 : 100 dilution of anti-Orai1 (D-15) (sc-74776; Santa Cruz Biotechnology, Santa Cruz, CA, USA) or 1 : 100 dilution of anti-STIM1 (610954; BD Biosciences, 1 : 100) as primary antibodies and the appropriate secondary antibodies tagged with Alexa Fluor-FITC or Alexa Fluor-rhodamine. Nuclei were stained with 5 *μ*g/mL 4′,6-diamidino-2-phenylindole (DAPI; D3571, Invitrogen, Molecular Probes, Eugene, Oregon, USA). The coverslips were rinsed, mounted with fluorescent mounting medium (S3023, Dako, Glostrup, Denmark), and cells were visualised on a confocal microscope (LSM 710 controlled with Zen software; Carl Zeiss, Jena, Germany).

### 2.5. ELISA Assay

WT and* STIM1*^−*/*−^ microglia were seeded into 6-well plates at 2 × 10^6^ cells/well and incubated at 37°C overnight. Then, cells were treated with or without 100 ng/mL LPS and incubated at 37°C in an incubator enriched with 5% CO_2_ atmosphere for 24 h. The next day, supernatants were assayed for levels of TNF-*α* and IL-6 using an ELISA kit (430901 and 431301, resp.; BioLegend, San Diego, CA, USA) according to the manufacturer's instructions. Cytokine levels in LPS-activated microglia were analysed for 30 min (at 5-min intervals) using the kinetic method and a VersaMax spectrophotometer plate reader (Molecular Devices, Sunnyvale, CA, USA) at 650 nm.

### 2.6. Phagocytosis Assay

WT and* STIM1*^−*/*−^ microglia were seeded into 12-well plates at 3 × 10^5^ cells/well and incubated at 37°C overnight. Then, cells were treated with or without 100 ng/mL lipopolysaccharides from* Escherichia coli* 0111:B4 (L4391, Sigma Aldrich, St. Louis, MO, USA) and incubated at 37°C in an incubator enriched with 5% CO_2_ atmosphere for 24 h. LPS-activated microglia were treated with BioParticles Fluorescent Particles (conjugated with Alexa Fluor 488; E-13231, Invitrogen, Molecular Probes, Eugene, Oregon, USA) and incubated at 37°C in an incubator enriched with 5% CO_2_ atmosphere for 2 h, and then cells were rinsed three times with PBS. To quench the particle fluorescence, cells were treated with 0.4% trypan blue stain (15250-061, Gibco, Life Technologies, Carlsbad, CA, USA) at room temperature for 2 min, and then cells were rinsed three times with PBS. Cells were fixed with 4% paraformaldehyde in PBS at room temperature for 10 min, and then rinsed three times with PBS. Nuclei were stained with 5 *μ*g/mL DAPI. Cells were visualised on an inverted fluorescence microscope (IX73-F22PH, Olympus), and fluorescence intensity was quantified with MetaMorph software (Molecular Devices, Sunnyvale, CA, USA). Flow cytometry experiments were conducted as follows. WT and* STIM1*^−*/*−^ microglia were seeded into 12-well plates at 3 × 10^5^ cells/well and incubated at 37°C overnight. Then, cells were treated with or without 100 *μ*M UDP and incubated at 37°C in an incubator enriched with 5% CO_2_ atmosphere for 20 min. UDP-activated microglia were treated with BioParticles Fluorescent Particles as described above. Cells were rinsed three times with PBS, treated with 0.05% trypsin-EDTA (25300-062, Gibco, Life Technologies, Carlsbad, CA, USA) at 37°C for 10 min in a CO_2_-enriched incubator, and then cells were harvested by centrifugation at 2,000 rpm at 4°C for 5 min. Supernatant was removed, 500 *μ*L ice-cold serum-free medium was added, and cells were resuspended. Cells were held on ice and analysed by flow cytometry using BD FACSVerse™ (BD Biosciences, Franklin Lakes, NJ, USA) and a total gated cell number of 10,000 cells. Flow cytometry data were analysed with FlowJo™ software (Treestar, Ashland, OR, USA).

### 2.7. In Vitro Chemotaxis Assay

Analysis of microglial migration was performed in triplicate using chemotaxis chambers (101-8, Neuro Probe, Gaithersburg, MD, USA) as previously described [13, 21]. Polycarbonate filters with 8-*μ*m pores were coated with 10 *μ*g/mL fibronectin (F0895, Sigma Aldrich, St. Louis, MO, USA) in PBS at room temperature for 1 h. The lower chambers were filled with 29 *μ*L of serum-free DMEM-F12 media containing 50 *μ*M ATP. The dry fibronectin-coated filter was installed to separate the lower chambers from the upper chambers. Then, WT and* STIM1*^−*/*−^ microglia were seeded into the upper chambers at 4 × 10^4^ cells/well in 20 *μ*L serum-free DMEM-F12, and chambers were incubated at 37°C in an incubator enriched with 5% CO_2_ atmosphere for 15, 30, 60, and 90 min. After incubation, the chemotaxis chambers were disassembled and filters were removed. Microglial cells in the lower chambers were stained with tomato lectin (conjugated with DyLight 594, 1 : 200 dilution, Vector Labs, Carlsbad, CA, USA), nuclei were stained with 5 *μ*g/mL DAPI at room temperature for 10 min, and then cells were rinsed three times with PBS. Cells that had migrated to the lower chambers were visualised and counted using an inverted fluorescent microscope (IX73-F22PH, Olympus, Tokyo, Japan). The total number of cells stained with DAPI was quantified using MetaMorph software (Molecular Devices, Sunnyvale, CA, USA).

### 2.8. Microinjection of Embryonic Mouse Brain In Vivo

We used microinjection to target specific regions of embryonic mouse brain in vivo as previously described [22]. Nanoliter 2000 microinjector and capillary glass (3.5 inches) (WPI, Sarasota, FL, USA) were used as injection tools. Timed pregnant* STIM1*^*+/*−^ hetero female mice carrying embryonic day 17 (E17) embryos were anesthetised with Isoflurane and placed on a heating pad. Embryonic mice in utero were injected with 30 nL of LPS mixed with 4% methylene blue (66720; Sigma Aldrich, St. Louis, MO, USA). The final concentration was 12 ng of LPS per one embryo in utero. The mother was sutured and recovered on a warming pad for 1 h, and then moved back to her home cage. After 24 h, the mother was reanesthetized with Isoflurane and the abdominal incision was reopened to approach the embryos. After the embryos were taken out of the utero, the mother was inevitably sacrificed. Embryonic whole brains were fixed with 10% formalin at 4°C overnight. Then, the tissue was transferred to 3 M sucrose in PBS solution and incubated at 4°C until the tissue sinks to the bottom of the tube (a couple of hours). This sucrose-infiltration is necessary for tissue cryopreservation. The fixed embryo brain tissue was used for cryosectioning 10 *μ*M thick slices, and embryo tails were used for genotype analyses. Sections of embryonic WT and* STIM1*^−*/*−^ brains were stained in blocking solutions with anti-Iba1 (1 : 200 dilution; 019-19741, Wako, Osaka, Japan), an antibody specific for microglia, and a secondary antibody labelled with Alexa Fluor 488 (Invitrogen, Molecular Probes, Eugene, Oregon, USA) and then rinsed three times with PBS. Nuclei were stained with 5 *μ*g/mL DAPI. After rinsing, coverslips were mounted with fluorescent mounting medium (S3023, Dako, Glostrup, Denmark) and visualised on a confocal microscope (LSM 710 controlled with Zen software, Carl Zeiss, Jena, Germany). Three brains of WT and* STIM1*^−*/*−^ embryonic mice were used for the experiments. The brain slices were illustrated using a tile scan mode to obtain the larger images of the entire brain. Among the larger images, the images which showed the injected legion with methylene blue were selected for counting the aggregated microglia and the highest number of aggregating microglia were used for summary. The total numbers of cells in lesion and counterpart areas were measured by computer vision using MetaMorph software.

### 2.9. Microglial Phagoptosis of Neurons

Normal neurons were cultured in the bottom dish of transwell plates to induce damage by secreted molecules from the LPS-activated microglia localized in the upper transwell dishes. Using this coculture system for 48 hours, the neuron became damaged according to Neher et al., [23, 24]. WT and* STIM1*^−*/*−^ microglia were treated with LPS for 24 hours to full activation. Activated WT and* STIM1*^−*/*−^ microglia were treated directly in contact with the damaged neuron to allow phagoptosis, for six hours. The same number of microglia in the same number of damaged neurons were used, and no activated microglia treated neurons were used as mocks. After six hours, all cells were fixed with 3.7% formaldehyde in the PBS, then immunostaining was performed as above. Cells were also stained with DAPI (for nuclei, blue), tomato lectin (for microglia, Rhodamine-red), and MAP (for neuron, FITC-green), respectively. The phagocytosis activity was relatively analysed with the portion of the fully enlarged microglia cell number (>35 *μ*m^2^) from the total number of the activated microglia (>25 *μ*m^2^). The relative neuron survival rate from the microglial phagoptosis was summarized by calculating each MAP (FITC) staining intensity from the remaining neurons in the five randomly selected images from the fields. No microglia treated only neurons were used for mocks to compare their phagoptosis activities.

### 2.10. Statistical Analyses

Data are presented as the means ± standard error of the mean. Statistical analysis was performed with Student's *t*-test or with analysis of variance (ANOVA), followed by Tukey's multiple comparison tests using the GraphPad Prism software package (version 5.0), as appropriate. *p* < 0.05 was considered statistically significant.

## 3. Results

### 3.1. LPS-Stimulated Phagocytosis and Cytokine Secretion in WT and* STIM1*^−*/*−^ Microglia

**Figure 1:**
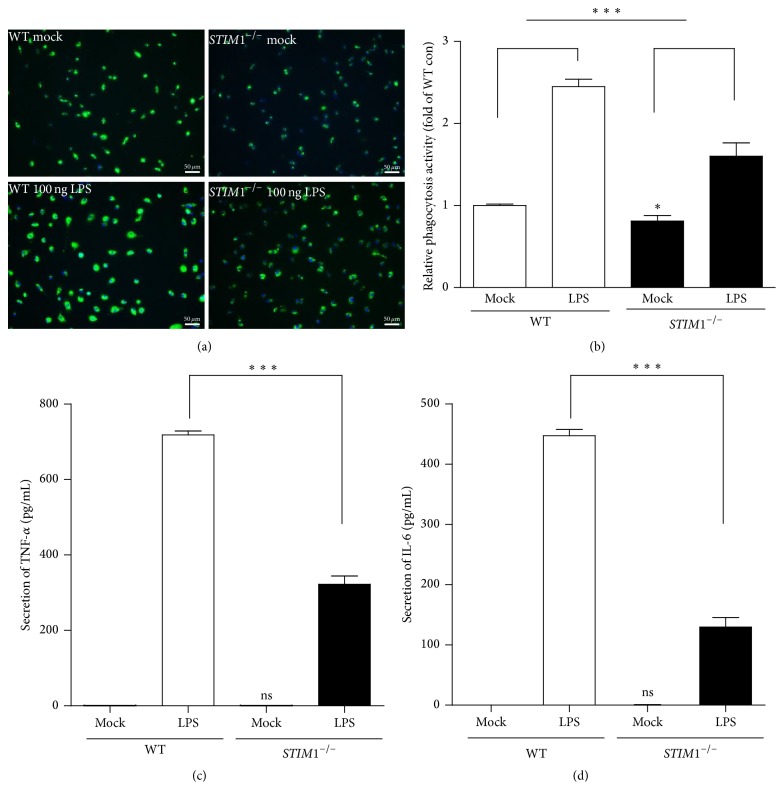
Lipopolysaccharide-induced phagocytosis and cytokine secretion in wild-type and* STIM1*^−*/*−^ microglia. (a, b) Representative images of image-based phagocytosis measurement using phagocytic engulfment of FITC bead in WT and* STIM1*^−*/*−^ microglia. Summary of five independent phagocytosis measurements. Seven images were used for phagocytosis measurements and then depicted as a bar graph. ^*∗∗∗*^*p* ≤ 0.0001 in LPS-induced fold-induction of WT; ^*∗*^*p* ≤ 0.005 in WT mock, *n* = 7. (c, d) Comparisons of LPS-induced cytokine secretion activity: TNF-*α* secretion (c) and IL-6 secretion (d) were compared in WT and* STIM1*^−*/*−^ microglia. *p* ≤ 0.0001, *n* = 4, where *n* is independent ELISA experiment; ns, no significant difference from WT mock.

The basal characteristics of WT and* STIM1*^−*/*−^ microglia were confirmed before using them for further experiments (Supplementary Figures  1~3 in Supplementary Material available online at https://doi.org/10.1155/2017/8158514). LPS is a potent immune activator of microglia, which binds and activates TLR4 receptor signalling [25]. STIM1 effect on microglial immune function, phagocytic activity, and cytokine secretion was compared in WT and* STIM1*^−*/*−^ microglia. To compare phagocytosis activity in WT and* STIM1*^−*/*−^ microglia, resting and LPS-stimulated (100 ng/mL for 24 h) phagocytotic activities were tested with engulfment of* E. coli* opsonised FITC-labelled bioparticles. Microglial cells were treated with bioparticle beads for 2 h. Then, free beads and attached beads on the cell surface were quenched by trypan blue, and cells were visualised using fluorescence microscopy (Figures 1(a) and 1(b)).* STIM1*^−*/*−^ microglia under resting-state conditions showed a slight reduction in phagocytotic activity. In WT cells, LPS treatment for 24 h increased phagocytic activity by approximately 2.5-fold, but* STIM1*^−*/*−^ microglia had smaller increases in phagocytic activity. These results indicate that the LPS-stimulated greater phagocytotic activity was more obviously found in WT microglia. Next, we compared LPS-induced secretion of TNF-*α* and IL-6 cytokines (Figures 1(c) and 1(d)). The results show that* STIM1*^−*/*−^ microglia secreted less than or equal to half of the amount of TNF-*α* and IL-6, as that secreted by WT microglia. These data indicate that STIM1 depletion reduces phagocytotic activity and cytokine section in the microglia under LPS-stimulation conditions.

### 3.2. UDP-Induced Phagocytosis Is Impaired in* STIM1*^−*/*−^ Microglia

**Figure 2:**
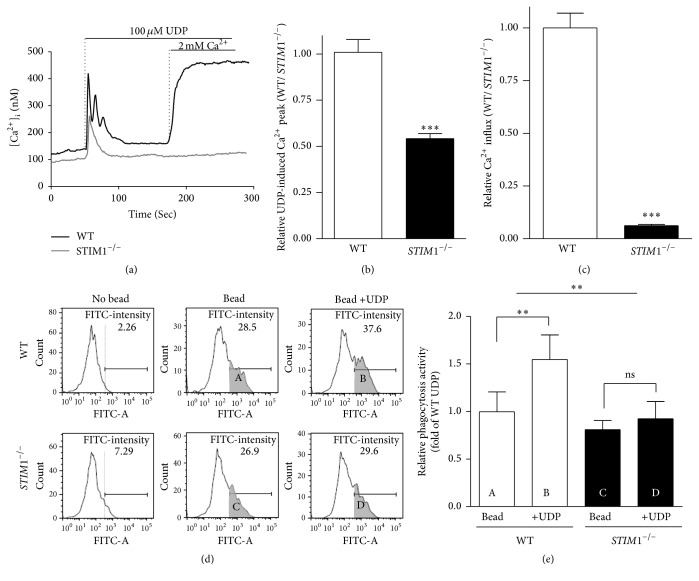
Comparison of UDP-induced Ca^2+^ signalling and phagocytosis in wild-type and* STIM1*^−*/*−^ microglia. (a) Representative curves of UDP-induced [Ca^2+^]_i_ mobilization in WT and* STIM1*^−*/*−^ microglia; 100 *μ*M UDP induced ER-Ca^2+^ efflux, whilst store depletion induced Ca^2+^ influx. (b) Relative UDP-induced Ca^2+^ transient release from ER. ^*∗∗∗*^*p* ≤ 0.0001, *n* = 15. (c) Comparison of UDP-induced SOCE activity determined by the slope of Ca^2+^ influx. ^*∗∗∗*^*p* ≤ 0.0001, *n* = 15. (d) Representative flow cytometry images of the phagocytosis measurements. FITC intensities of phagocytic bead engulfment with or without 100 *μ*M UDP were measured at 20 min. Grey region indicates the population of bead-engulfed microglia. (e) Summary of FACS from three independent phagocytosis experiments. Geometric mean represents the relative phagocytosis activity in each set. ^*∗∗*^*p* ≤ 0.001 in comparisons of bead engulfment in WT microglia.

Phagocytosis of microglia is activated by UDP through P2Y_6_, metabotropic receptor that is coupled to the Gq-protein [10]. RT-PCR analysis indicated that microglia expresses P2X_3_, P2X_4_, and P2X_7_ receptors and all types of P2Y receptors (Supplementary Figure  4). In particular, the expression of P2Y receptors were somewhat increased in* STIM1*^−*/*−^ microglia, which implies that the compensatory up-regulation of P2Y receptors might exist to cope with the reduced Ca^2+^ influxes in* STIM1*^−*/*−^ microglia. We measured UDP-induced [Ca^2+^]_i_ in WT and* STIM1*^−*/*−^ microglia (Figure 2(a)). In* STIM1*^−*/*−^ microglia, 100 *μ*M UDP induced a transient increase in [Ca^2+^]_i_ but no sustained Ca^2+^ influx (Figures 2(a)–2(c)). This result indicates that UDP-induced calcium influx is almost disappeared in* STIM1*^−*/*−^ microglia. To more precisely test the microglial phagocytosis activity, we tested the phagocytotic engulfment of* E. coli* opsonised, FITC-labelled bioparticles using FACS (Figures 2(d) and 2(e)). This method enabled discrimination of the UDP-induced phagocytosis peak from the basal phagocytosis peak. After phagocytotic bead engulfment for 20 min with or without UDP, microglia were detached by trypsin, and collected cells were counted by FACS.  Figure 2(d) shows the representative FACS images of WT and* STIM1*^−*/*−^ microglia. Phagocytosis activity of WT and* STIM1*^−*/*−^ microglia after 20 min of UDP treatment is presented in Figure 2(e). UDP-induced phagocytosis in* STIM1*^−*/*−^ microglia was severely defected compared to that in WT. These data indicate that STIM1 is critically important in UDP-induced phagocytosis.

### 3.3. Chemotactic Migration Is Severely Defective in* STIM1*^−*/*−^ Microglia

**Figure 3:**
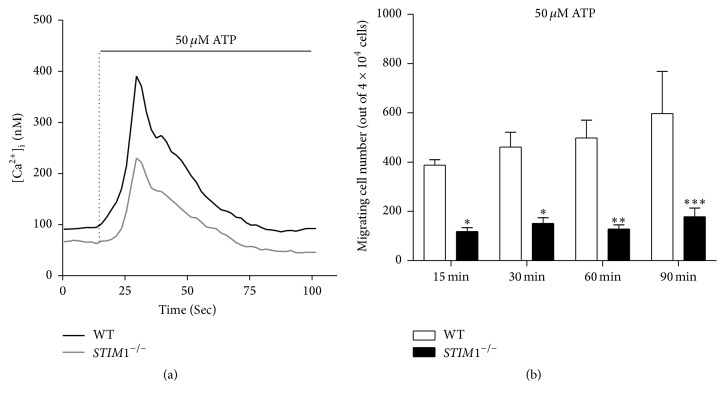
Chemotactic migration in wild-type and* STIM1*^−*/*−^ microglia. (a) Treatment with 50 *μ*M ATP-induced [Ca^2+^]_i_ changes in 2 mM Ca^2+^ media. (b) Time-dependent migration toward 50 *μ*M ATP determined in chemotaxis chambers. Cells migrating toward ATP were fixed, stained with DAPI, and counted after 15, 30, 60, and 90 min. The numbers of migrating cells at each time point were determined from six independent experiments and are depicted graphically. ^*∗*^*p* ≤ 0.05, ^*∗∗*^*p* ≤ 0.001, and ^*∗∗∗*^*p* ≤ 0.0001 versus the corresponding control (*n* = 6, where *n* is the number of independent experiments).

ATP is a chemoattractant of microglia, and ATP concentrations of approximately 50–100 *μ*M activate several purinergic receptors [12, 26]. First, we monitored [Ca^2+^]_i_ by measuring Fura-2-AM fluorescence under the same conditions that were used for chemotactic migration experiments.* STIM1*^−*/*−^ microglia had a reduced Ca^2+^ evocation signal than WT microglia (Figure 3(a)). Next, chemotactic migration to ATP in chemotaxis chambers in WT and* STIM1*^−*/*−^ microglia was compared. The number of cells migrating into the ATP chamber after 15, 30, 60, and 90 min were counted after DAPI staining. WT microglial migration was time-dependent and increased with longer incubation times. By contrast,* STIM1*^−*/*−^ microglia did not show a time-dependent increase in migration, and the number of cells migrating into the ATP chamber was one-third of that for WT microglia. These results clearly indicate that* STIM1*^−*/*−^ microglia have defective chemotactic migration to ATP (Figure 3(b)). These data show that STIM1 is crucial for microglial chemotactic migration activity.

### 3.4. Peripheral/Central Localization of Orai1 Is Controlled by STIM1 upon UDP Stimulation

**Figure 4:**
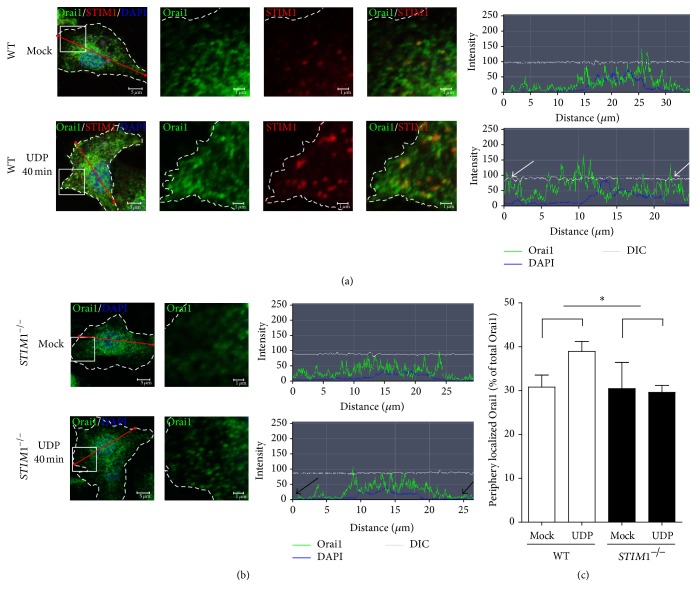
Retarded peripheral localization of Orai1 in* STIM1*^−*/*−^ microglia upon UDP stimulation. UDP induces movement of Orai1 (green) and STIM1 (red) in WT microglia (a) and* STIM1*^−*/*−^ microglia (b), respectively. Treatment with 100 *μ*M UDP for 40 min increased Orai1 localization at the cell surface throughout the cell border in WT(a) and* STIM1*^−*/*−^ microglia (b). Insets were magnified following separated three (a) or one image (b). Orai1-FITC-intensity profiles in red lined regions were shown in each of the rightmost images. The cell border is indicated by dotted lines. Clustered Orai1 in peripheral regions of WT microglia were indicated by white arrows, and the absence of Orai1 in peripheral regions of* STIM1*^−*/*−^ microglia was indicated by black arrows. (c) Intensity of surface Orai1 in cell periphery was compared with the UDP condition. Intensity of surface Orai1 in cell periphery was determined by the intensity of ROI with 0.65 *μ*m away from the cell edges, using MetaMorph software.

We supposed that Orai1 is a microdomain indicator of [Ca^2+^]_i_, since Ca^2+^ influxes through Orai1 which can change [Ca^2+^]_i_ underneath the plasma membrane and build a microdomain of [Ca^2+^]_i_. To test whether the peripheral localization of Orai1 was controlled by STIM1, we compared localization of Orai1 under UDP treatment which induce acute phagocytic movement in WT and* STIM1*^−*/*−^ microglia [27]. At the resting state, Orai1 was merely found at the cell periphery. After 40 min of UDP treatment, Orai1 clusters in the peripheral region were significantly increased, and some of them were colocalized with STIM1 clusters in WT microglia (Figure 4(a)). Such clusters of Orai1 in peripheral regions were not observed in* STIM1*^−*/*−^ microglia (Figure 4(b)). These results suggested that STIM1 regulates Orai1 localization upon UDP treated conditions, and it might imply that STIM1 controls [Ca^2+^] microdomains in the cell periphery that requires for local Ca^2+^ signalling for phagocytic or chemotactic migration movements.

### 3.5. Microglial Aggregation in LPS-Injected Lesions Was Abolished in* STIM1*^−*/*−^ Embryonic Brain

**Figure 5:**
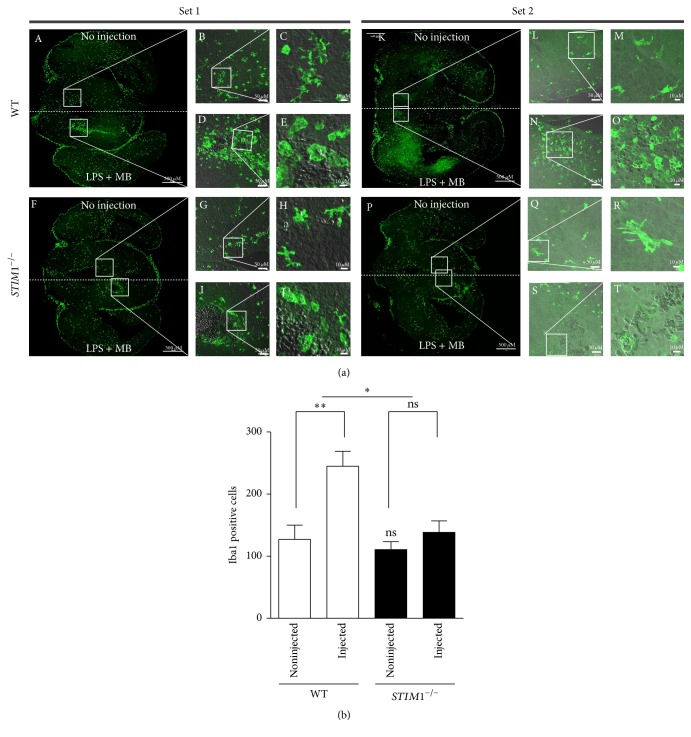
In vivo behaviour of microglia in lipopolysaccharide-injected brains of wild-type and* STIM1*^−*/*−^ embryonic mice. (a) Two experimental sets representing microglial aggregation at the site of injection of LPS and methylene blue (MB) in WT and* STIM1*^−*/*−^ mice brain. No LPS injection (upper right hemisphere) and LPS-injected (lower left hemisphere) are indicated in low-magnification images of immunostaining with Iba1 antibody (A, F, K, and P; bar = 500 *μ*m). White boxed regions are twofold magnifications ((B, D, G, I, L, N, Q, and S; bar = 50 *μ*m) and (C, E, H, J, M, O, R, and T; bar = 10 *μ*m)). Merged images of green Iba1-FITC and grey-DIC are shown in B–E, G–J, L–O, and Q–T; microglia are shown in green and methylene blue particles are shown in grey. Resting ramified microglia (C, H, M, and R) were identified in the noninjected hemisphere, and the stimulated amoeboid shapes of microglia engulfing methylene blue particles (E, J, O, and T) were identified only in the LPS + MB injected region. (b) Comparison of aggregated microglial numbers at the LPS injection site. The numbers of microglia at the LPS + MB injection hemisphere and noninjected hemisphere were counted. ^*∗*^*p* ≤ 0.05; ^*∗∗*^*p* ≤ 0.01; ns, no significant difference from WT mock. Data are representative of three experiments.

To test the in vivo behaviour of microglia, we injected 30 nL LPS plus methylene blue mix into brains of 17-day-old embryos impregnated in* STIM1*^*+/*−^ female mice mated with* STIM1*^*+/*−^ male mice. Methylene blue was used to label the injection site in the tissue slices. At 24 h after injection, each embryo brain was harvested and their genotypes were checked. WT and* STIM1*^−*/*−^ brains were sectioned, and the sections containing lesions labelled with methylene blue were selected and stained with Iba1 to visualise microglia. Two sets of representative WT and* STIM1*^−*/*−^ brain tissue sections at different magnifications are presented in Figure 5(a). In WT brain, Iba1-labelled microglia aggregated at the LPS-methylene blue injected lesion, but the counterpart region of the noninjected hemisphere displayed normal ramified microglia. High-magnification images of both hemispheres clearly show their distinct morphologies as ramified resting state, and amoeboid activated state. In* STIM1*^−*/*−^ brain tissue, the LPS-methylene blue injected lesion was not crowded with microglia, and the microglia localized in the lesion showed similar amoeboid shapes as those of WT. We calculated the total microglial cell number in the whole-brain slices, and did not find any significant difference in the cell numbers as shown in mocks in* STIM1*^−*/*−^ brain tissue. These results also were observed when the number of cells after primary culture of each mouse was counted. However,* STIM1*^−*/*−^ brain slices had definitely reduced numbers of Iba1 positive microglia aggregated in LPS-injected lesions (Figure 5(b)), which indicates that* STIM1*^−*/*−^ microglia have a defect in migration activity toward the legion. These in vivo data indicate that* STIM1*^−*/*−^ microglia have severe defects in chemotactic migration activity in brain environment.

### 3.6. Neuron Phagocytosis Activity Is Attenuated in* STIM1*^−*/*−^ Microglia

**Figure 6:**
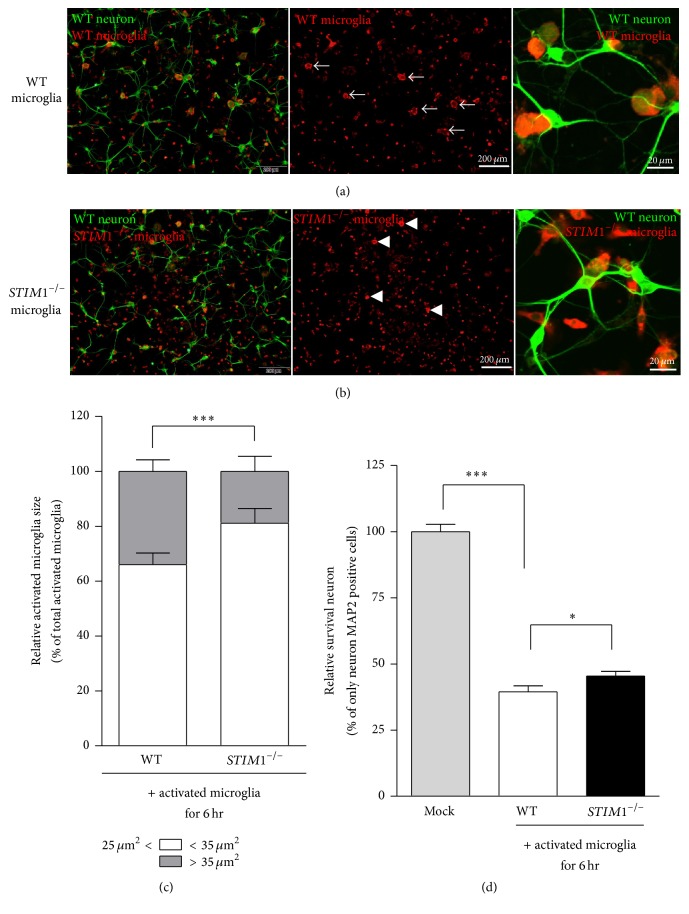
Decreased neuron phagoptosis activity of* STIM1*^−*/*−^ microglia. (a) Neuron phagoptosis by WT microglia was shown by immunostaining of neuron and microglia after damaging neuron and LPS-activated WT microglia (bar = 200 *μ*m). Normal neurons were stained with MAP specific antibodies (green), and WT microglia were stained with tomato lectins (red). The second image shows enlarged microglia indicated via arrows. The third image shows highly magnified images of the activated microglia engulfing neurons, so MAP staining was found inside of the microglia stained with tomato lectin. (b) Similar image to the above, but used LPS-activated* STIM1*^−*/*−^ microglia which coexisted with neurons. The second image shows some enlarged* STIM1*^−*/*−^ microglia indicated with arrows. The third image shows highly magnified images showing the activated microglia near the neurons. (c) The relative size of the activated WT and* STIM1*^−*/*−^ microglia indicated via bar graph. 25 *μ*m^2^ was determined as the minimum size of activated microglia, and the number of cells bigger than 35 *μ*m^2^ was assigned as the “fully enlarged microglia engulfing neuron,” which is significantly small in* STIM1*^−*/*−^ microglia population. ^*∗∗∗*^*p* ≤ 0.001. *n* ≥ 100; *n* = cell number from 3 independent experiments. (d) The relative neuron survival rate was analysed using MAP2 staining intensity of the remaining neuron after 6 hr phagoptosis, in which images were delivered from five randomly selected fields of leftmost images. Each of the phagoptosis activities was compared with the total MAP2 intensity of the only neuron which was without phagoptosis by activated microglia. ^*∗∗∗*^*p* ≤ 0.001, ^*∗*^*p* ≤ 0.05, *n* ≥ 15, and *n* = 5 images from 3 independent experiments.

To compare the microglial phagocytosis activities, we tested the phagocytic activity of WT and* STIM1*^−*/*−^ microglia in the condition of direct contact with damaged cortical neurons. Since the LPS treatment without microglia does not cause damage of neuron [24], we applied the coculture systems using WT microglia with 100 ng/mL LPS treatment on transwell inserts, and the primary cultured cortical WT neurons on the bottom of the plate for two days to prepare for the phosphatidylserine- (PS-) exposed WT neuron [24]. After two days, the transwell insert was removed and the medium was also washed out, and preactivated (100 ng/mL LPS for 24 h) WT or* STIM1*^−*/*−^ microglia were directly added to PS-exposed damaged neuronal cells [23]. After 6 h, the cortical neuron and microglia cells were fixed and cells were indicated with corresponding antibodies (Figures 6(a) and 6(b)). We analysed each size of tomato lectin positive microglia cells, and counted the fully enlarged microglia engulfing neurons. The total number of fully enlarged microglia (>35 *μ*m^2^) out of the total number of activated microglia (>25 *μ*m^2^) was significantly higher in WT microglia (Figure 6(c)). These data indicated that LPS-activated WT microglia had higher neuron phagocytosis activities than* STIM1*^−*/*−^ microglia. Furthermore, the neuronal survival rate from phagoptosis by* STIM1*^−*/*−^ microglia was much higher than that of WT microglia (Figure 6(d)). All of the data suggests that STIM1 is important in phagoptosis activities of microglia.

## 4. Discussion

One of the most crucial signalling pathways during immune cell differentiation is sustained calcium elevation in the cytosol, which is mediated primarily by SOCE [14]. The significance of SOCE was demonstrated in studies using pharmacological inhibitors and depletion of extracellular calcium with calcium chelators, but it has not been fully evaluated in microglial cells under completely blocked conditions until* STIM1* was identified [15, 20]. Complete depletion of SOCE was necessary to determine whether the complete SOCE depletion abolished immune response activation of immune responses, which is mediated by sustained [Ca^2+^]_i_ increase in response to several agonists. A complete depletion of SOCE was achieved in purified lymphocytes of* STIM1*^−*/*−^ mice, including mast cells, T cells, platelets, neutrophils, and microglia [20, 28–32]. Our previous using siRNA for STIM1 was not able to completely deplete all STIM1 protein, so the effect of residual STIM1 in activated immune function of microglia could not be ruled out [33]. In this study, we evaluate the in vitro and in vivo effects of STIM1-regulated SOCE on microglial immune functions including phagocytosis, cytokine secretion, and chemotactic migration. Not only the Ca^2+^ signal which changes the profile or efficiency of gene expression, but also the Ca^2+^ signal which acutely regulates the function such as UDP-induced phagocytosis and ATP-induced chemotaxis was significantly decreased in STIM1 depletion condition.

STIM2 was less effective to activate SOCE than STIM1 in T cells [14, 28] and microglia [32]. Michaelis et al. recently reported that* STIM2*^−*/*−^ microglia displayed SOCE activation by store depletion and nucleotide stimulation, compared with the complete absence of SOCE in* STIM1*^−*/*−^ microglia [32]. Western blot analyses and [Ca^2+^]_i_ measurements indicated that STIM1 protein expression was greater than that of STIM2 (Supplementary Figure  1B). SOCE was essentially abolished after CPA-induced Ca^2+^ depletion from ER in* STIM1*^−*/*−^ microglia (Supplementary Figures  2A and 2D) in spite of the presence of STIM2 (Supplementary Figure  1B). These results indicate that STIM1 is the major regulator of SOCE in microglia.

Important roles of STIM1 in differentiation of active microglial status were proved by attenuated immune function of activated microglia treated with LPS. LPS-induced activated microglia showed increased phagocytosis activity and cytokine secretion of TNF−*α* and IL-6, but those were significantly reduced in* STIM1*^−*/*−^ microglia. The sustained high [Ca^2+^]_i_ via Ca^2+^ influx causes the change of Ca^2+^ concentration of nucleus which yields the change of gene expression for fully differentiation [14]. These reduced chronic immune function of* STIM1*^−*/*−^ microglia might be caused by the reduced Ca^2+^ influx and lower [Ca^2+^]_i_ status, which abolishes the increase of nuclear Ca^2+^ concentration to change the gene expression for fully differentiation. Moreover, the crucial role of STIM1 in acute movement was proved by UDP-induced phagocytosis and ATP-stimulated chemotaxis. The UDP-induced phagocytosis is relatively faster function which demands the cytoskeletal movement governed by local Ca^2+^ signal in the cell periphery [10]. The markedly impaired UDP-induced phagocytosis in* STIM1*^−*/*−^ microglia seems to be caused by local increased Ca^2+^ concentration right after the influx. Similarly, ATP-stimulated microglial chemotaxis and migration were greatly reduced in* STIM1*^−*/*−^ microglia subjected to chemotaxis assays. Time-dependent ATP-stimulated cell migration was essentially abolished in* STIM1*^−*/*−^ microglia (Figure 3(b)). These defects might be related with the retarded movement of Orai1 in* STIM1*^−*/*−^ microglia (Figure 4). Orai1 pores can constitute a [Ca^2+^] microdomain underneath the plasma membrane through the Ca^2+^ influx. Attenuated movements of Orai1 in* STIM1*^−*/*−^ microglia implied that STIM1 is critical for the organization of [Ca^2+^] microdomains in microglia.

This in vitro result was confirmed in in vivo studies. The cell migration to the injured site was severely impaired in the brain environment (Figure 5) and damaged neuron phagoptosis was much more retarded in* STIM1*^−*/*−^ microglia (Figure 6). Injured cells release nucleotides such as ATP, which is a known chemotactic attractant and an activator of purinergic receptors such as P2X_4_ and P2Y_12_, and rapid cell migration is essential for microglial recruitment to the site of brain lesions [12, 13]. Although the severe impairment of cell migration in* STIM1*^−*/*−^ embryonic brain is not only due to defective chemotaxis of* STIM1*^−*/*−^ microglia, since the environment of* STIM1*^−*/*−^ brains is also differs from that of WT brains in our experimental conditions. However, defective chemotaxis of* STIM1*^−*/*−^ microglia in vitro and retarded phagocytosis activity toward damaged neurons strongly suggest that STIM1 is essential for regulating chemotactic migration and phagocytosis activated by chemical cue or damaged neurons.

Cell migration is coordinated by Ca^2+^ signalling, in part through local Ca^2+^ signalling in the cell leading edge [9]. Previous reports have identified STIM1 localization in podosome [34], phagosome [34], and cell-matrix adhesion at the front of migrating cells [9]. Recent work showed that receptor tyrosine kinase signalling and phospholipase C signalling are restricted to the front of the migrating endothelial leader cells, which triggers local Ca^2+^ pulses, local depletion of ER-Ca^2+^, and local STIM1 activation; these cascades orchestrate pulsatile retraction and adhesion of the leading cell front [9]. Another study reported that STIM1 localization is juxtaposed to ER and phagosomes, which generate Ca^2+^ hotspots that boost phagocytosis [7]. Our results are in agreement with these previous studies that STIM1-regulated local Ca^2+^ influx in the leading focal adhesion microdomain is important for the function mediated by local cytoskeletal rearrangement, such as UDP-induced rapid phagocytosis and ATP-induced chemotactic migration. Notably, STIM1 depletion had a stronger adverse effect on the rate of UDP-induced rapid phagocytosis and ATP-induced chemotactic migration (Figures 2(d), 2(e), and 3(b)) than that on activation of LPS-induced phagocytotic activity (Figures 1(a) and 1(b)) and LPS-induced cytokine secretion (Figures 1(c) and 1(d)). These data might imply that the role of STIM1 in local Ca^2+^ microdomains is more critical than the role of STIM1 in [Ca^2+^]_i_ change through the cells.

A recent study indicated that STIM1 inhibition in lymphocytes had beneficial effects for anaphylactic responses [20], ischemic brain infarction [30], and autoimmune disease [31]. The current model of neuronal degeneration considers that inflamed microglia have detrimental effects that lead to neuronal loss [2]. The retarded LPS-induced activation of* STIM1*^−*/*−^ microglia implied that inhibition of STIM1 can attenuate the magnitude of flammability of microglia. Our result showed that* STIM1*^−*/*−^ microglia has reduced phagoptosis activity toward damaged neurons (Figure 6). These results suggest that STIM1 can be used as a new therapeutic target to prevent the excessive immunological microglial function and thereby delay the progression of neurodegenerative diseases.

In conclusion, we found that* STIM1*^−*/*−^ microglia showed reduced cytokine secretion and opsonin-dependent phagocytosis in LPS condition. More importantly, UDP-induced phagocytosis and ATP-stimulated chemotactic migration were severely impaired in* STIM1*^−*/*−^ microglia in vitro and in vivo. These results suggest that STIM1-mediated store-operated calcium entry is also important for the regulation of global [Ca^2+^]_i_ changes, but it is indispensable for regulating local [Ca^2+^] microdomains in murine microglia.

## Supplementary Material

Supplement Figure 1. mRNA and protein Expression of SOCE elements in primary cultured microglia from wild-type and* STIM1*^−*/*−^ mice. Supplement Figure 2. Cyclopiazonic acid (CPA) induced [Ca^2+^]i changes in WT and* STIM1*^−*/*−^ microglia. Supplement Figure 3. Thapsigargin induced Orai1 and STIM1 co-localization in WT microglia. Supplement Figure 4. Expression of purinergic receptors in primary cultured microglia from wild-type and* STIM1*^−*/*−^ mice. Supplement Table 1. Primers used in Supplement Figure 4.

## Abbreviations

SOCE: Store-operated calcium entry
[Ca^2+^]_i_: Intracellular Ca^2+^ concentration
SOC: Store-operated channel
STIM1: Stromal interaction molecule 1
STIM2: Stromal interaction molecule 2
UDP: Uridine diphosphate
ATP: Adenosine triphosphate
LPS: Lipopolysaccharide
CPA: Cyclopiazonic acid
TG: Thapsigargin
TNF-*α*: Tumour necrosis factor-*α*
IL-6: Interleukin-6
siRNA: Small interfering RNA
WT: Wild-type
MB: Methylene blue
TLR4: Toll-like receptor 4
NFAT: Nuclear factor of activated T cells
ER: Endoplasmic reticulum.
