# Supplementary material for: UDP-Induced Phagocytosis and ATP-Stimulated Chemotactic Migration Are Impaired in STIM1−/− Microglia In Vitro and In Vivo
Source: Mediators Inflamm. 2017 Feb 15;2017:8158514. doi: 10.1155/2017/8158514 (PMC5331284; doi:10.1155/2017/8158514)
Supplement: Supplementary file 1 — Supplement Figure 1. mRNA and protein Expression of SOCE elements in primary cultured microglia from wild-type and STIM1−/− mice. Supplement Figure 2. Cyclopiazonic acid (CPA) induced [Ca2+]i changes in WT and STIM1−/− microglia. Supplement Figure 3. Thapsigargin induced Orai1 and STIM1 co-localization in WT microglia. Supplement Figure 4. Expression of purinergic receptors in primary cultured microglia from wild-type and STIM1−/− mice. Supplement Table 1. Primers used in Supplement Figure 4. [file 8158514.f1.pdf]

Supplement Figure 1. mRNA and protein Expression of SOCE elements in primary cultured microglia from wild-type and *STIM1*$^{-/-}$ mice.

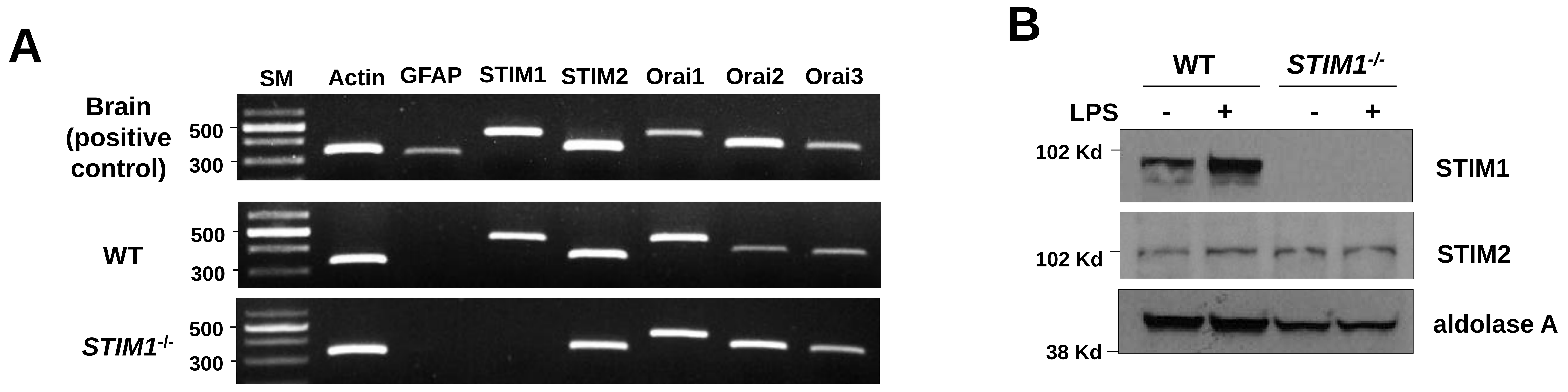

Supplement Figure 2. Cyclopiazonic acid (CPA) induced $[Ca^{2+}]i$ changes in WT and *STIM1*$^{-/-}$ microglia.

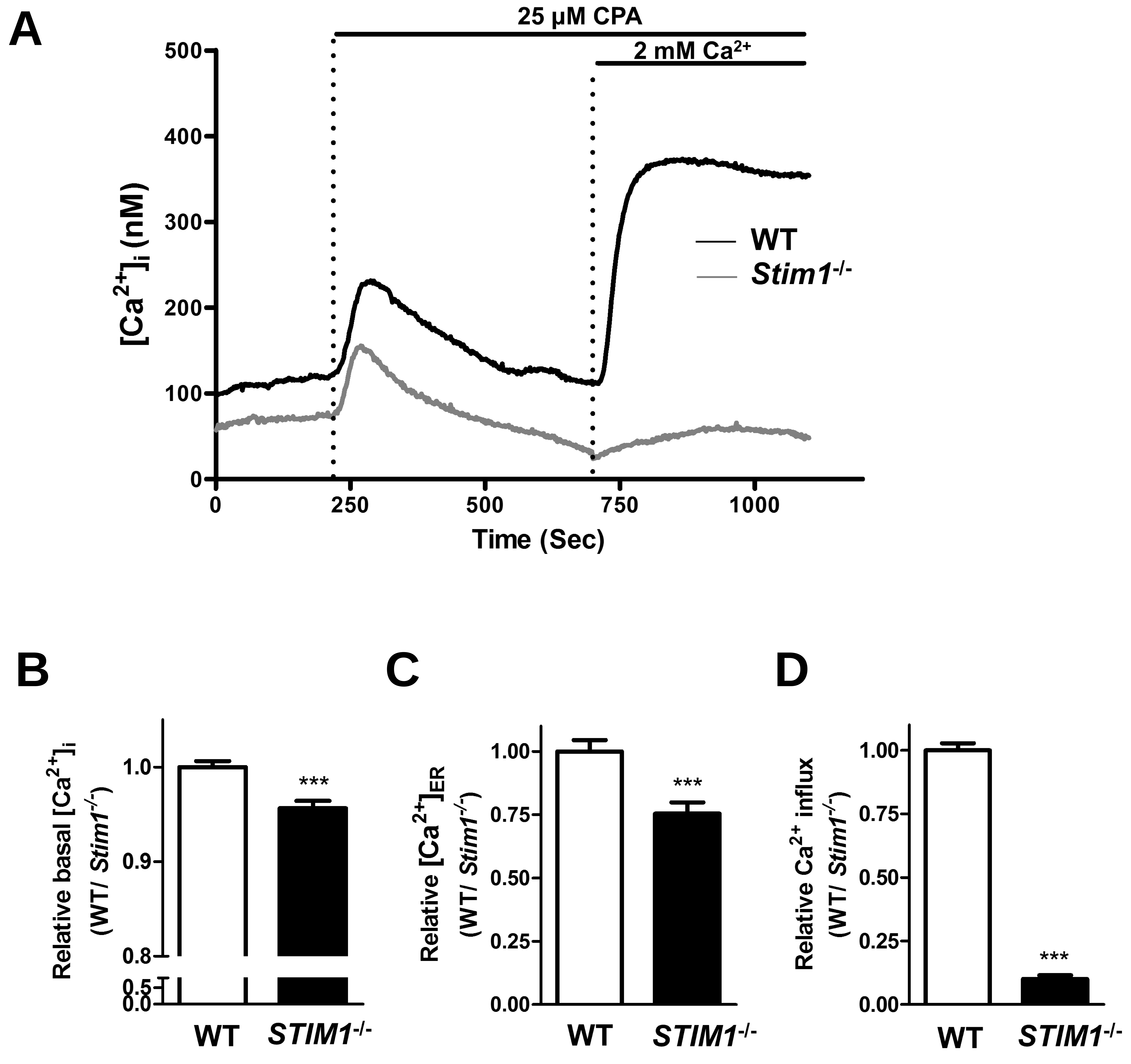

Supplement Figure 3. Thapsigargin induced Orai1 and STIM1 co-localization in WT microglia.

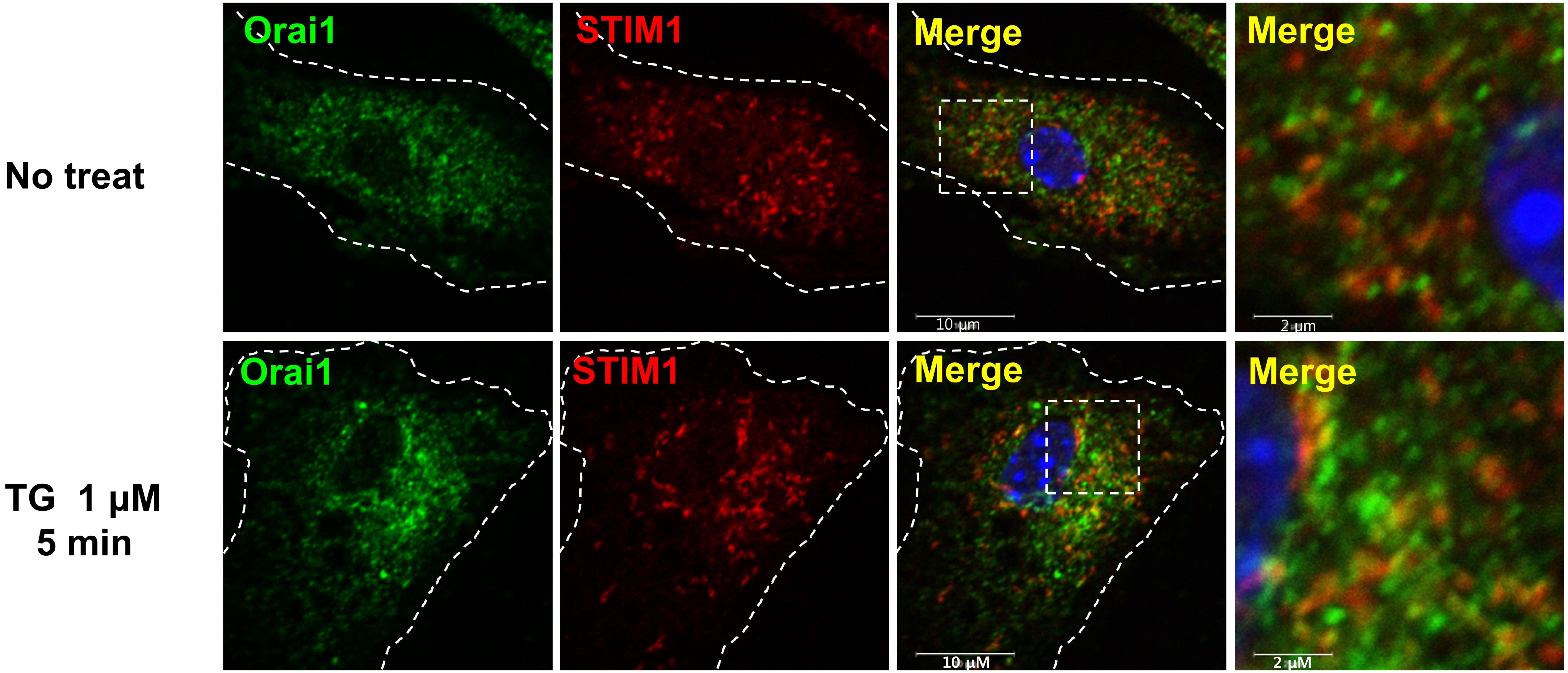

Supplement Figure 4. Expression of purinergic receptors in primary cultured microglia from wild-type and *STIM1*$^{-/-}$ mice.

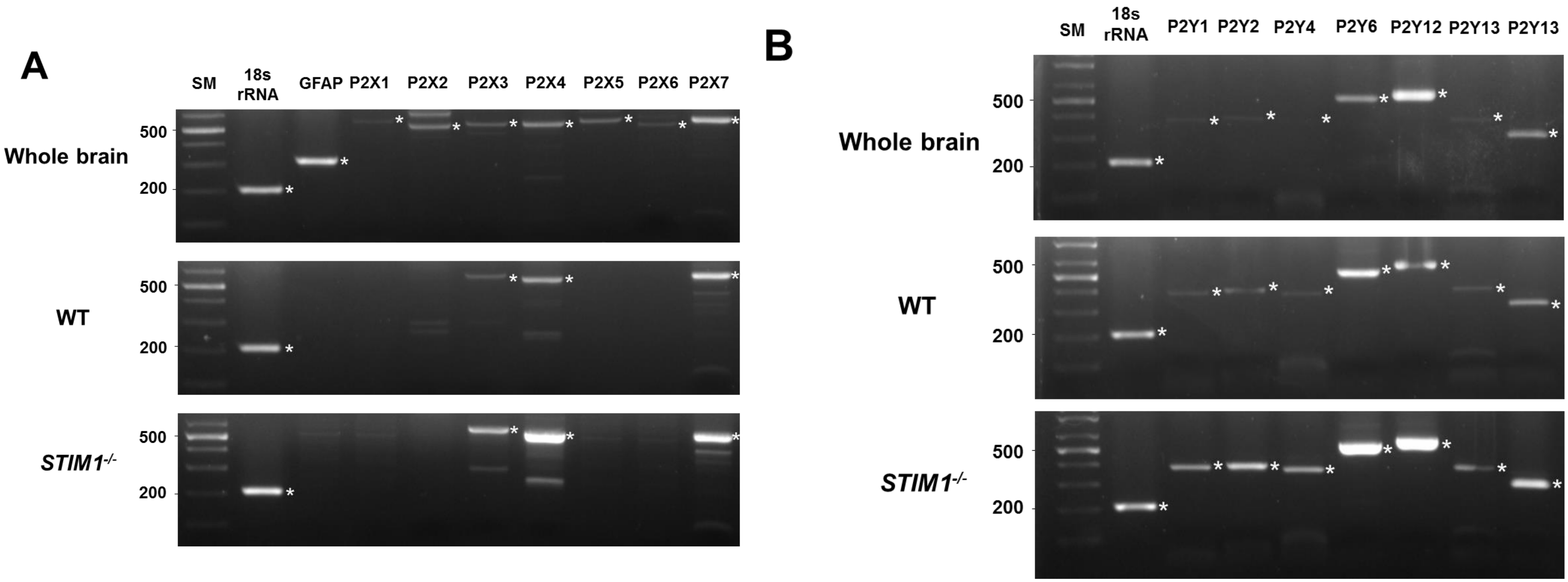

Supplement Table 1. Primers used in supplement figure 4.

Gene name	Primers	Product size
P2X1	S, 5′-GAAACTCAAGGGCTTGGCTGTGAC-3′,<br>AS, 5′-TTCTCAGCAAGGCTTCGGAAGTCC-3′	546 bp
P2X2	S, 5′-TCGGAACACAAAGTGTGGGACGT-3<br>AS, 5′-CCCTTGTGTGCCAGTTCTGTGAAG-3	515 bp
P2X3	S, 5′-GCCGTTCAGCTGCTGATTATCTCC-3<br>AS, 5′-TGGAAGCGGCACTTCTTTATGTCC-3	533 bp
P2X4	S, 5′-ATGGCAGGCTGCTGCTCCGT-3<br>AS, 5′-AGCTGGCGTTGGCACACC-3	531 bp
P2X5	S, 5′-TAAAGCTGCCTCCCACTGCAAC-3<br>AS, 5′-GCAAGATCTGTGATGGCTTCATGTTC-3	554 bp
P2X6	S, 5′-AGGAACTGGGAACATGGCTTCTGC-3<br>AS, 5′-TCCTGTGGGTTCCGTTGAACAC-3	497 bp
P2X7	S, 5′-ATGCCGGCTTGCTGCAGC-3<br>AS, 5′-TCGGCGCTCCTCAAGAGT-3	557 bp
P2Y1	S, 5′-CCAGTTCTACTACCTGCCG-3<br>AS, 5′-CACACCAGCACGCTGAC-3	381 bp
P2Y2	S, 5′-GTTGTGCCTGAACGTCG-3<br>AS, 5′-CGCTGGTGGTGACGAAG-3	386 bp
P2Y4	S, 5′-CTGCAAGTTCGTCCGCTTTC-3<br>AS, 5′-GGTCGATACAGTCGCCGG-3	369 bp
P2Y6	S, 5′-ATGGAGCAGGACAATGGCACC-3<br>AS, 5′-ACACAGTGCGGTTGCGCT-3	529 bp
P2Y12	S, 5′-ATGGATGTGCCTGGTGTCAACACC-3<br>AS, 5′-CGTGCCAAACTAGACCAAACTCTGAC-3	580 bp
P2Y13	S, 5′-ATCAACACCACTGGGATGCA-3<br>AS, 5′-CCTGAACGGCATGATGATC-3	387 bp
P2Y14	S, 5′-CCACCACAGACCCTCCAAAC-3<br>AS, 5′-GAACACGATGCTGACGTACAT-3	320 bp
18S rRNA	S, 5′-TGCGCCGCTAGAGGTGAAATTC-3<br>AS, 5′-GGCATCGTTTATGGTCGGAACTAC-3	200 bp
GFAP	S, 5′-TGATGGAGCTCAATGACCG-3<br>AS, 5′-CATCTGCCTCCTGTCTATACGC-3	315 bp
